# Protection against inhalation anthrax by immunization with *Salmonella enterica serovar Typhi* Ty21a stably producing protective antigen of *Bacillus anthracis*

**DOI:** 10.1038/s41541-017-0018-4

**Published:** 2017-06-15

**Authors:** B. Kim Lee Sim, Minglin Li, Manuel Osorio, Yun Wu, Tint T. Wai, Johnny W. Peterson, Eric R. James, Sumana Chakravarty, Lixin Gao, Rui Xu, Natasha KC, Richard E. Stafford, William S. Lawrence, Linsey A. Yeager, Jennifer E. Peel, Satheesh K. Sivasubramani, Ashok K. Chopra, Svetlana Filippova, Stephen L. Hoffman

**Affiliations:** 1 Protein Potential LLC, Rockville, MD 20850 USA; 2 U.S. Food and Drug Administration (FDA), Center for Biologics Evaluation and Research (CBER), Silver Spring, MD 20993 USA; 3 Department of Microbiology and Immunology, Galveston National Laboratory, Center for Biodefense and Emerging Infectious Diseases, and Sealy Center for Vaccine Development, University of Texas Medical Branch, Galveston, TX 77555 USA; 4 Sanaria Inc, Rockville, MD 20850 USA

## Abstract

The national blueprint for biodefense concluded that the United States is underprepared for biological threats. The licensed anthrax vaccine absorbed vaccine, BioThrax, requires administration of at least 3–5 intramuscular doses. The anthrax vaccine absorbed vaccine consists of complex cell-free culture filtrates of a toxigenic *Bacillus anthracis* strain and causes tenderness at the injection site and significant adverse events. We integrated a codon-optimized, protective antigen gene of *B. anthracis* (plus extracellular secretion machinery), into the chromosome of the licensed, oral, live-attenuated typhoid fever vaccineTy21a to form Ty21a-PA-01 and demonstrated excellent expression of the gene encoding protective antigen. We produced the vaccine in a 10-L fermenter; foam-dried and vialed it, and characterized the dried product. The vaccine retained ~50% viability for 20 months at ambient temperature. Sera from animals immunized by the intraperitoneal route had high levels of anti-protective antigen antibodies by enzyme-linked immunosorbent assay and anthrax lethal toxin-neutralizing activity. Immunized mice were fully protected against intranasal challenge with ~5 LD_50_ of *B. anthracis* Sterne spores, and 70% (7/10) of vaccinated rabbits were protected against aerosol challenge with 200 LD_50_ of *B. anthracis* Ames spores. There was a significant correlation between protection and antibody levels determined by enzyme-linked immunosorbent assay and toxin-neutralizing activity. These data provide the foundation for achievement of our ultimate goal, which is to develop an oral anthrax vaccine that is stable at ambient temperatures and induces the rapid onset of durable, high-level protection after a 1-week immunization regimen.

## Counterterrorism: creating a safe, stable, easily stored anthrax vaccine

A vaccine candidate for anthrax infection shows promise for improving preparedness for a biological attack. *Bacillus anthracis*, the bacterium responsible for anthrax is a top-tier bioterrorism agent due to its high lethality and spore stability. The current FDA-approved anthrax vaccine and other vaccine candidates in development lack ease of preparation, have short shelf lives and adverse effects. B. Kim Lee Sim of Protein Potential LLC and her collaborators combined key *B. anthracis* genetic material into an existing typhoid vaccine. The vaccine vector possesses high stability, a strong safety record, and offers long-term protection after oral administration, which Sim’s group hopes to preserve in their candidate anthrax vaccine. The team showed that their hybrid vaccine conferred excellent protection in rabbits and a short vaccination regimen, and suggest further studies into its suitability for human vaccine studies.

## Introduction

A bipartisan blue ribbon panel on biodefense recently concluded that the United States is not prepared for a biological attack, and urges that new advances in scientific technology and new biodefense vaccines be developed to prevent mass casualties in the future due to bioterrorism attacks.^1, 2^ Anthrax is caused by the spore-forming bacterium, *Bacillus anthracis*. When anthrax spores are transmitted by the respiratory route, inhalation anthrax rapidly progresses to a septic shock-type syndrome with a high case-fatality rate if antibiotic treatment is delayed.^3, 4^ Anthrax spores are physically stable and can be readily processed into an easily distributable and inhalable powder. *B. anthracis* is on the Center for Disease Control and Prevention list of Tier-1bioterrorism agents.^5^

The primary virulence factors of *B. anthracis* include the poly-D-glutamate capsule, and three toxin subunits, namely protective antigen (PA), lethal factor (LF), and edema factor (EF) that form two binary toxins. Lethal toxin (LT) is composed of PA and LF, and edema toxin (ET) consists of PA and EF.^6^ PA binds to host cell receptors, is enzymatically processed and oligomerizes, and then forms a molecular pore that selectively allows for the translocation of EF and LF into the cytosol^7–9^ to manifest their effects in the host cells.

Neutralizing antibodies against PA alone protect humans, rabbits, and non-human primates against anthrax,^10–20^ and form the basis of the current US-licensed AVA vaccine (anthrax vaccine adsorbed, BioThrax) and UK-licensed AVP vaccine (anthrax vaccine precipitated). The AVA vaccine is an aluminum hydroxide-adsorbed, PA-containing culture filtrate prepared from a non-encapsulated, toxinogenic strain of *B. anthracis* (V770-NP1-R). For pre-exposure prophylaxis, AVA is administered intramuscularly in five doses at 0, 1, 6, 12 and 18 months, with annual boosting.^21^ A shortened regimen of two doses did not completely eliminate spores in a non-human primate spore challenge model.^22^ Due to the prolonged immunization regimen, this is not an optimal vaccine for pre-exposure prophylaxis, or for post-exposure therapy. Moreover, it is difficult to achieve high levels of compliance in large populations, due to the prolonged dosing schedule and reactogenicity. Currently, the stockpiling of sufficient AVA for emergency use is a challenge, because of its relatively short 4-year shelf life and its requirement for storage at 2–8 °C.^21^ Thus, various expression systems, immunogens, adjuvants, and delivery vehicles have been explored in efforts to develop alternative anthrax vaccines.^10, 23–28^

Live attenuated *Salmonella* spp. have been an attractive alternate platform for constructing an anthrax vaccine. Such a vaccine can be self-administered orally. Immunization by the oral route elicits both mucosal and systemic immune responses. Moreover, *Salmonella* vaccines can stimulate both humoral and cellular immune responses. Previously published studies using attenuated *S.* Typhimurium^29–32^ or *S.* Typhi^33–36^ expressing PA to immunize mice or non-human primates, either alone or together with a boost of recombinant PA, demonstrated excellent immunogenicity or accelerated immune response in the case of priming, and protection against inhalation anthrax. Among these *Salmonella* expression platforms, Ty21a (Vivotif) has the unique advantage that it is one of the only licensed oral, live-attenuated bacterial vaccine strains. For preventing typhoid fever, Ty21a is administered during 1 week and protects humans for at least 7 years,^37, 38^ and has an unrivaled safety record over a period of 25 years.^38–45^ There has never been a reported case of bacteremic dissemination of Ty21 after administration to more than 200 million recipients.^46^ Further, there are no reports of post-vaccination inflammatory arthritis (e.g., Reiter’s syndrome) with Ty21a, a potential problem with other live attenuated vectors including nontyphoid *Salmonella*, *Shigella*, and *Yersinia*.^47^ Ty21a can be foam-dried, which provides temperature stabilization and a potential shelf life of 5–10 years,^48^ and Ty21a has been shown to be an excellent candidate vector for foreign antigen delivery, including PA.^34–36^

Most of the previously reported proof-of-concept studies expressed PA from a plasmid,^30, 32–36^ which is an unstable expression technology due to plasmid loss and consequential loss of antigen expression. A prior study in animals inferred that even a stabilized plasmid can be lost once the bacterium is administered to humans.^35^ We stably integrated the gene encoding PA into Ty21a creating a vaccine strain Ty21a-PA-01, which we foam-dried, used it to immunize mice and rabbits by the intraperitoneal route (IP) and showed protection against lethal doses of inhaled *B. anthracis* Sterne and Ames spores.

## Results

### Recombineering of the gene encoding PA (stable integration)

Previously, it was demonstrated that Ty21a carrying a plasmid expressing a codon-optimized, proteolysis-resistant PA that was mutated at the furin and chymotrypsin cleavage sites, fused to the *hlyA* secretion signal and the *hlyB-D* extracellular secretion apparatus, and controlled by an optimized constitutive *lpp* promoter, was immunogenic and protective in mice.^34^ Compared with native PA,^29, 30, 33, 35, 36^ this form of PA provided an extra level of safety, as N-terminal processing of PA is required for toxin activation and PA multimeric pore formation.^49^ However, this plasmid was unstable.^34^ Therefore, the PA expression cassette was recombinationally inserted into the silent Vi gene region of Ty21a.^50^ The selectable marker was then deleted from the insert, resulting in a final, markerless chromosomal integrant. Previous assessments of Ty21a carrying the cloned PA encoding gene on a plasmid showed that, although the strain expressed and produced high levels of PA, the plasmid was unstable in the absence of antibiotic selection. Only 5% of Ty21a cells retained the plasmid after 25 generations of growth.^34^ In contrast, the chromosomally integrated clone was confirmed to be 100% genetically stable for high levels of PA production after >100 generations of growth (colony immunoblot and western blot). This clone was designated Ty21a-PA-01.

### Seed bank generation and characterization of the recombinant *S*. Typhi Ty21a

We generated a seed bank designated as SB 02APR2013 Ty21a-PA-01. Characterization of this seed bank demonstrated that all microbiological, immunological, phenotypic, and genetic properties were as expected (Table 1). The integrity of the 6,130 bp PA expression cassette antigenic insert was verified by DNA sequencing. Furthermore, the expected chromosomal insertion location at the *tviE* locus was verified by sequencing.

**Table 1:** Characterization of Ty21a-PA-01 seed bank

Assay	Method	Vial 1	Vial 2	Ty21a	Ty2	*E. coli*
Microbiological	API 20 E	*S*. Typhi	*S*. Typhi	*S*. Typhi	*S*. Typhi	*E. coli*
	Colony appearance on Bromothymol blue agar ( + 1% galactose)	Blue	Blue	Blue	Yellow	Blue
Biochemical	Minimal media + cysteine + thiamine + tryptophan	No growth	No growth	No growth	Growth	No growth
	Minimal media + cysteine + thiamine + tryptophan + valine + isoleucine	Growth	Growth	Growth	Growth	Growth
	Heat stress at 55 °C for 20 min	Sensitive	Sensitive	Sensitive	Resistant	Sensitive
	Oxidative stress in 0.3% H_2_O_2_ for 20 min	Sensitive	Sensitive	Sensitive	Resistant	Sensitive
	Galactose (1%) induced bacteriolysis	Sensitive	Sensitive	Sensitive	Resistant	ND
Immunological	9,12 O-antigen agglutination	+	+	+	+	ND
	Vi antigen agglutination	−	−	−	+	ND
	PA Expression (colony and western blot)	+	+	−	ND	ND
Genetic	16 S rDNA sequence	Identical to Ty21a	Identical to Ty21a	Ty21a	Ty2	ND
	*galE* DNA sequence	Identical to Ty21a	Identical to Ty21a	Ty21a (T367C; C442Δ)	Ty2 (wild type)	ND
	Chromosomally integrated PA expression-secretion cassette (PCR)	+	+	−	−	ND

Two random vials from the seed bank were characterized.^38^ Ty21a (Vivotif), *S. enterica* serovar Typhi strain Ty2, and *E. coli* strain HB101 were controls

Ty21a-PA-01 was subjected to growth in a bioreactor using CY medium at 5–10 L scales. The pH was maintained between 7–8.3, because in preliminary studies it provided the best stability.^51^ We routinely achieve growth characteristics of ~1 × 10^10^ CFU/mL (final OD_600nm_ ~ 9.0).

### Foam-drying and stability

To achieve optimization of foam drying, several different foam-drying protective agents (FPAs) in various combinations and concentrations were evaluated for the effect on dried cell viability and residual water content, an indicator of long-term survivability. Foam-dried product, obtained with an optimized FPA formulation, was subjected to a temperature stability analysis at 4 °C, ambient temperature (20–25 °C), and 37 °C over a period of 20 months (Table 2). To date, foam-dried Ty21a-PA-01 has been stable at 4 °C and ambient temperature for 20 months (Table 2). This foam-dried product showed viability profiles (Table 2) and water content (~5–6% throughout the study period) comparable to those previously reported when foam-drying of Ty21a resulted in a temperature-stabilized formulation that survived at acceptable levels at 25 °C for 12 months and had a predicted shelf life of 5–10 years at 4 °C.^48^

**Table 2:** Stability characteristics of foam-dried Ty21a-PA-01

Time point (months)	Viable count (× 10^6^ CFU ± SD/mg foam-dried product) at respective storage temperatures		
	4 °C	Ambient	37 °C
0	N/A	57.7 ± 9.6	N/A
1	75.4 ± 4.7	89.4 ± 9.1	7.2 ± 2.4
3	63.1 ± 11.6	38.8 ± 13.1	3.6 ± 0.9
15	56.8 ± 5.6	24.8 ± 5.4	N/A
20	66.9 ± 15.5	27.4 ± 5.2	N/A

The water content (~5 to 6%) was stable throughout the study period. Ambient temperature was 20–25 °C

*N/A* not available or not tested

### Antibody responses in mice

**Fig. 1:**
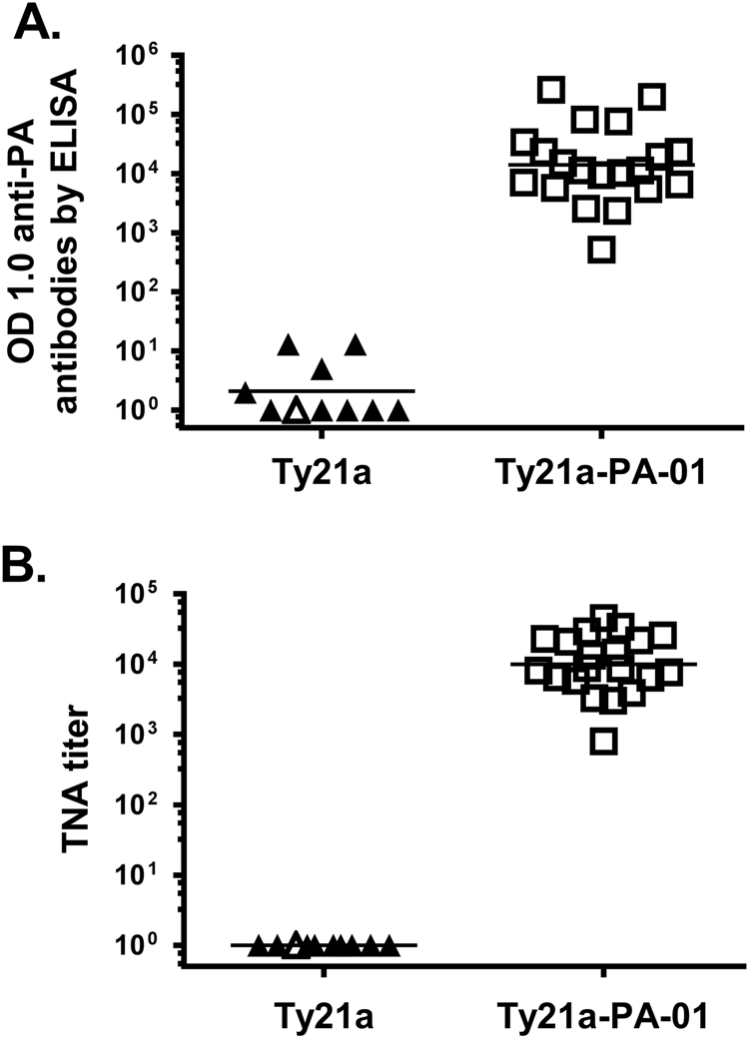
Antibodies in mice immunized with Ty21a-PA01. Mice were vaccinated with 5 × 10^7^ CFU of Ty21a (*triangles*) or Ty21a-PA-01 (*squares*) by intraperitoneal (IP) injection at 2-week intervals. Sera collected at 2 weeks after dose 3 were assayed for anti-PA IgG by ELISA (**a**) and anthrax toxin neutralization activity (TNA) (**b**). Antibody responses by ELISA are reported as the serum dilution at which the optical density was 1.0 (OD 1.0), and TNA titers as the last serum dilution at which there was 50% protective activity against killing of macrophages (J774A.1) by LT. *Filled symbols* represent mice that were not protected in the spore challenge study and open symbols represent those that were protected (see Fig. 3). The geometric mean of each group is shown as a horizontal line. Antibody responses between the two groups were analyzed by Mann–Whitney U test (*p = *1.1 × 10^−5^ and 8.2 × 10^−6^ for ELISA and TNA, respectively)

Sera from immunized mice were collected 2 weeks after dose 3 (time of challenge) and assessed for antibodies to PA by enzyme-linked immunosorbent assay (ELISA) (Fig. 1a), and toxin-neutralizing activity (TNA) (Fig. 1b). For mice immunized with Ty21a-PA-01, the geometric mean OD 1.0 (serum dilution at which the optical density was 1.0) of anti-PA antibodies was 14,169 (range 518 - 263,960). The geometric mean TNA titer (serum dilution at which 50% of J774.1 cells were killed) was 9,942 (range 800–45,258). The control group immunized with Ty21a had a geometric mean OD 1.0 in the anti-PA ELISA of 2 (range 1–13) and a TNA titer of <1 in all mice. The difference in antibody responses between the immunized and controls groups was statistically significant (*p* = 1.1 × 10^−5^ and 8.2 × 10^−6^ for ELISA and TNA, respectively, Mann–Whitney *U* test, two-tailed).

### Antibody responses in rabbits

**Fig. 2:**
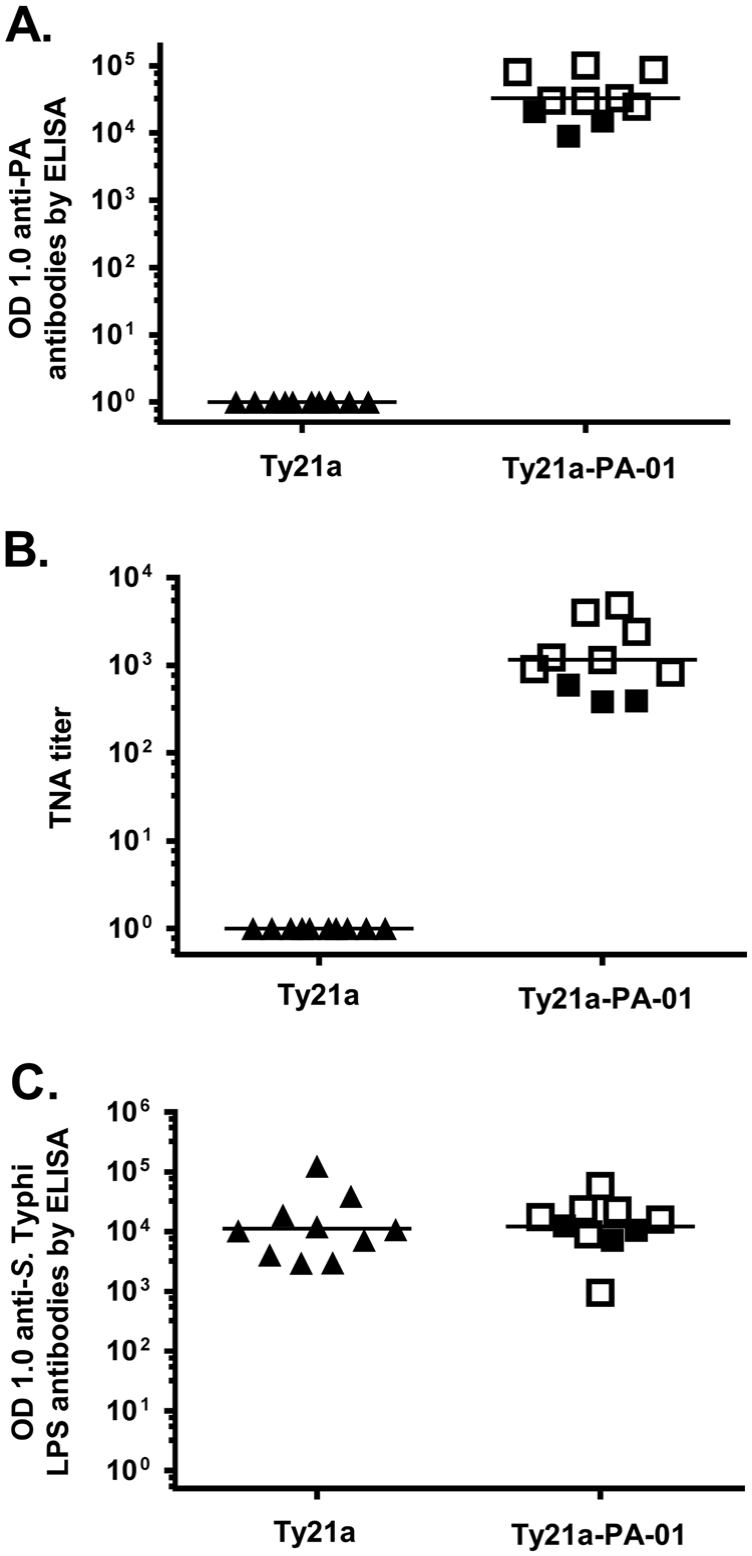
Antibodies in rabbits immunized with Ty21a-PA-01. Rabbits were vaccinated with 1 × 10^9^ CFU of Ty21a (*triangles*) or Ty21a-PA-01 (*squares*) by IP injection at 4-week intervals. Sera collected 2 weeks after dose 4 were assayed for anti-PA IgG by ELISA (**a**), anthrax toxin neutralization activity (TNA) (**b**), and anti-*S*. Typhi LPS IgG by ELISA (**c**). *Filled symbols* represent rabbits that were not protected in the spore challenge study and open symbols represent those that were protected (see Fig. 4). Geometric mean of each group is shown as a horizontal line. Anti-PA responses by ELISA and TNA responses, but not anti-*S*. Typhi LPS responses by ELISA, were significantly higher in the immunized as compared to control rabbits (*p* = 6.39 × 10^−5^, 6.39 × 10^−5^, and 0.53, respectively, by Mann–Whitney *U* Test). The geometric mean OD 1.0 of anti-PA antibodies by ELISA was 47,268 for the seven protected rabbits as compared to 14,107 for the three unprotected rabbits (*p* = 0.0167, Mann–Whitney *U* test). Likewise, the geometric mean TNA titer was 1,741 for the protected rabbits and 447 for the unprotected animals (*p* = 0.0167)

In immunized rabbits, sera were collected 2 weeks after dose 4 of the vaccine and assessed for antibodies to PA by ELISA (Fig. 2a), TNA (Fig. 2b), and *S*. Typhi lipopolysaccharide (LPS) (9 and 12 O antigens) by ELISA (Fig. 2c). For rabbits immunized with Ty21a-PA-01, the geometric mean OD 1.0 of anti-PA antibodies by ELISA was 32,887 (range 9,029–101,360). The geometric mean TNA titer was 1,158 (range 383–4,812). The control group of rabbits immunized with Ty21a had a geometric mean OD 1.0 in the anti-PA ELISA of <1 in all rabbits and a TNA titer of <1 in all rabbits. Rabbits immunized with Ty21a control or Ty21a-PA-01 produced similar levels of anti-*S*. Typhi LPS IgG antibodies (Fig. 2c). The geometric mean OD 1.0 by ELISA was 11,198 (range 2,970–123,520) for rabbits immunized with Ty21a control and 12,159 (range 953–58,140) for rabbits immunized with Ty21a-PA-01. The anti-PA antibody responses between the controls (Ty21a) and experimental (Ty21a-PA-01) groups were significantly different (*p* = 6.39 × 10^−5^ and 6.39 × 10^−5^ for ELISA and TNA, respectively). However, there was no significant difference between anti-*S*. Typhi LPS antibody responses in the control and experimental groups (*p = *0.53).

### Protective efficacy in mice

**Fig. 3:**
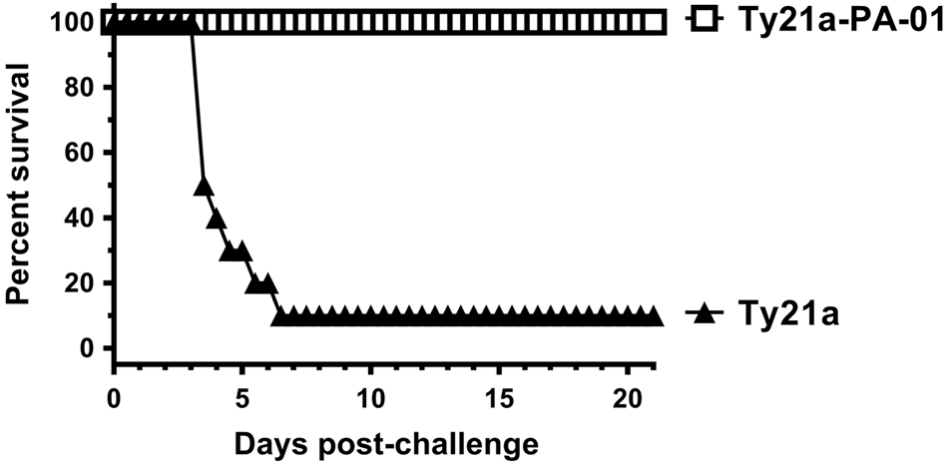
Ty21a-PA-01 protects mice from lethal spore challenge. Mice were immunized three times with 5 × 10^7^ CFU of Ty21a control (*filled triangles*) or Ty21a-PA-01 (*open squares*) by the IP route and were challenged two weeks after dose 3 with Sterne spores at a dose of 4.9 LD_50_ by intranasal installation and monitored twice daily for 21 days. Mean time to death (TTD) was calculated for the controls. As noted, 1 of 10 control mice immunized with Ty21a and 20/20 mice immunized with Ty21a-PA-01 survived (*p* = 2.2 × 10^−8^, Fisher’s exact test, two-tailed). For the nine control mice that died, the mean TTD was 4.2 ± 0.1 (mean ± SD) days

We challenged mice, immunized with Ty21a or Ty21a-PA-01, two weeks after dose 3 with five LD_50_ of Sterne spores by intranasal instillation. All of the animals (20/20) immunized with Ty21a-PA-01 and 1/10 control mice immunized with Ty21a survived (*p* = 2.2 × 10^−8^, Fisher’s exact test, two-tailed) and remained healthy throughout the 21-day monitoring period (Fig. 3). The mean time to death (TTD) in the control mice was 4.2 ± 0.1 (mean ± SD) days.

### Protective efficacy in rabbits

**Fig. 4:**
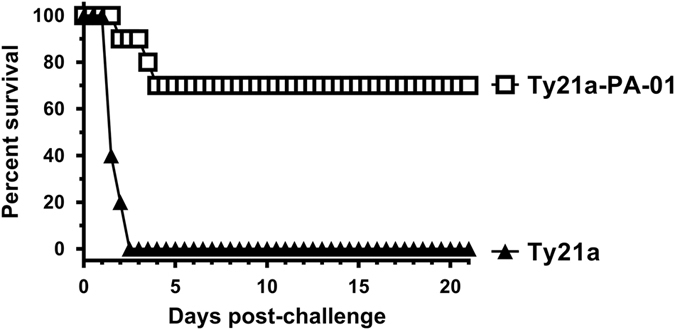
Ty21a-PA-01 protects rabbits from a high dose lethal Ames spore challenge. Rabbits were immunized with 1 × 10^9^ CFU of Ty21a control (*filled triangles*) or Ty21a-PA-01 (*open squares*) by the IP route four times at 4-week intervals. Two weeks after dose 4, rabbits were challenged with aerosolized Ames spores at a dose of 200 LD_50_ and monitored twice daily for 21 days. Mean TTD was calculated. None of ten control rabbits immunized with Ty21a survived; however, 7/10 rabbits immunized with Ty21a-PA-01 survived (*p* = 0.0031, Fisher’s exact test, two-tailed). All 10 control rabbits died in 1.8 ± 0.4 days, and the mean TTD for the 3 immunized rabbits that died was 3.2 ± 1 days (*p* = 0.039by Mann–Whitney *U* test, two-tailed)

We challenged rabbits that had been immunized with Ty21a-PA-01 or Ty21a, 2 weeks after dose 4 with 200 LD_50_ of spores from the fully virulent Ames strain by aerosol administration to the lungs. Seven of ten rabbits immunized with Ty21a-PA-01 and none of ten control rabbits immunized with Ty21a survived (*p* = 0.0031, Fisher’s exact test, two-tailed) (Fig. 4). All 7 surviving rabbits immunized with Ty21a-PA-01 remained healthy throughout the 21-day monitoring period. The ten control rabbits died in 1.8 ± 0.4 days and the mean TTD for the three Ty21a-PA-01-immunized rabbits that died was 3.2 ± 1 days, a statistically significant difference (*p* = 0.039, Mann–Whitney *U* test, two-tailed). All rabbits with a TNA titer > 825 and an anti-PA ELISA OD 1.0 titer > 24,651 survived (Fig. 2a and b). The three rabbits that were immunized with Ty21a-PA-01 and died had the lowest levels of LT neutralizing antibodies by TNA (Fig. 2b) and by anti-PA ELISA (Fig. 2a), but not against *S*. Typhi LPS (Fig. 2c). The geometric mean OD 1.0 of anti-PA antibodies by ELISA was 47,268 for the seven protected rabbits as compared to 14,107 for the three unprotected rabbits (*p* = 0.0167, Mann–Whitney *U* test, two-tailed). Likewise, the geometric mean TNA titer was 1,741 for the protected rabbits and 447 for the unprotected animals (*p* = 0.0167).

## Discussion

Our goal is to develop a well-tolerated, safe, highly protective anthrax vaccine that is stable at ambient temperatures and administered orally during 5–7 days in 3–4 doses. To achieve this goal, we have developed a recombinant live whole organism Ty21a attenuated typhoid vaccine stably expressing the *B. anthracis* PA gene, designated Ty21a-PA-01, by integrating the PA gene into the Ty21a chromosome.Ty21a-PA-01 is an advance over the previously reported protective Ty21a vaccine that expressed the PA gene via a plasmid, which is an unstable expression technology.^34–36^

We are the first to foam dry and vial a live bacterium expressing PA as an anthrax vaccine. We showed immunogenicity and protective efficacy of this foam dried and vialed product in mice and rabbits. Unlike some of the previously published reports,^33, 35^ Ty21a-PA-01 did not require a recombinant protein boost to achieve high levels of protection.

The virulence of *B. anthracis* is due to its toxins and a poly-D-glutamic acid capsule that is responsible for bacterial dissemination. A/J inbred mice are susceptible to death by exposure to *B. anthracis* strains producing capsule alone, regardless of the presence of toxins.^52^ To correlate the levels of anti-PA after immunization withTy21a-PA-01with protection against *B. anthracis*, we used the toxigenic, non-capsulated *B. anthracis* Sterne strain to challenge immunized mice.^10^ We found that protection against the Sterne strain correlated with anti-PA response. When administered to mice by IP injection, Ty21a-PA-01 was highly immunogenic, and protected 100% of the mice against a lethal nasal Sterne spore challenge dose.

We next immunized NZW rabbits, since they are considered to be a more stringent model of inhalation anthrax than mice. Using 200 LD_50_ of encapsulated, fully virulent Ames spores, 70%of the rabbits were protected (Fig. 4).

All of the rabbits developed antibodies to PA and exhibited significant toxin neutralizing activity. However, the three unprotected rabbits had the lowest levels of antibodies to PA and the lowest TNA titers (Fig. 2). There appeared to be a threshold level of antibodies required for complete protection. We infer that because Ty21a-PA-01 and Ty21a induced similar titers of anti-*Salmonella* O antigen (Fig. 2c), the protective efficacy of Ty21a-PA-01 against *Salmonella* Typhi will be equivalent to that provided by Ty21a. Thus, we argue that Ty21a-PA-01 can be developed as a bivalent vaccine against both typhoid fever and anthrax.

The immunization regimen in this study served as a proof-of-concept, and was not optimized. It may be possible to achieve 100% protection in rabbits by altering the dose of vaccine and/or spacing of doses to increase the level of protective anti-PA titers among low responding rabbits. Our next major step will be to assess immunogenicity and protective efficacy of Ty21a-PA-01 in non-human primates such as rhesus macaques.^53^ However, our goal is to immunize humans orally. Since *S*. Typhi (e.g. Ty21a) does not infect animals, there is no oral animal model available for Ty21a. That is why we immunized animals by the IP route. We also observed that in mice, a weeklong IP immunization regimen was inferior to immunizing IP at 2-week dosing intervals. Such data in animals have been and will continue to be important in vaccine development. However, because *S*. Typhi (e.g., Ty21a) does not infect animals, we cannot confidently extrapolate any results in animals to humans. Thus, we argue that human clinical trials are needed to adequately assess this vaccine.

With that in mind, we are first working to increase the potency of the vaccine by increasing the numbers of administered Ty21a-PA-01 bacteria that successfully reach the ileum where they are thought to invade M cells and induce protective immunity. One method to achieve this is to protect the vaccine from the low pH of gastric acidity by integrating acid stabilization genes into it. We have done this successfully with a Ty21a-*Shigella sonnei* O-antigen bivalent vaccine,^54, 55^ and intend to do so for Ty21a-PA-01.

We will then manufacture a master cell bank (MCB) in compliance with current Good Manufacturing Practices, and use the MCB to manufacture and foam dry the vaccine. In our current study, > 40% of original viable counts (CFU/mg) remained after 20 months storage of foam-dried product at 20–25 °C (ambient temperature), and there was no significant loss of viability when Ty21a-PA-01 was stored at 4 °C over our study period of 20 months (Table 2). We anticipate this vaccine may have a shelf life of 5–10 years at 4 °C and at least 12 months at ambient temperatures. In comparison, lyophilized Ty21a (Vivotif) lost 0.5log_10_ of titer during 1 week at 25 °C.^48^ These findings support the option to administer vaccine at ambient temperature during mass administration campaigns. Providing high-risk populations with a temperature stable vaccine for use by self-administration is also being considered.

The US Departments of Defense and Health and Human Services have spent enormous amounts of funds during the past decade to purchase and stockpile injectable anthrax vaccines that induce anti-PA protective antibodies after many months of immunization. We believe that in the event of a major disaster, our temperature-stabilized oral vaccine could be used for mass immunization of civilian, military, first-responders and medical personnel, and other vulnerable individuals of the population in a week-long immunization regimen, like the regimen used for Ty21a^56^ against typhoid fever.

Ty21a is safe and well tolerated when given orally over a 7-day immunization regimen, and protects humans against typhoid fever for at least 7 years.^38, 40^ We anticipate similar durable protection against both anthrax and typhoid fever with Ty21a-PA-01 in humans. From a broader perspective, we aim to use rTy21a to express multiple, stably integrated and expressed foreign antigens and establish rTy21a as a platform technology for creating orally administered vaccines against multiple pathogens that are stable at room temperature.^55^

## Materials and methods

### Bacterial strains and cell lines

Ty21a was commercially purchased (Vivotif, Crucell, Miami Lakes, FL). A seed bank was made in CY medium (1.2% yeast extract, 2% Hy-Case, 1.2% pepticase, 0.125% NaH_2_PO_4,_ 0.33% NaCl, pH 7.2, 0.2% glucose, and 0.005% galactose), which is currently utilized for Vivotif production.^46^ Spores of *B. anthracis* Sterne strain were purchased (Colorado Serum Co. Denver, CO) and washed free of saponin in sterile water, before quantitating by serial dilution and plating on 5% sheep blood agar plates. Spores of *B. anthracis* Ames strain were prepared in a 10 L bioreactor at pH 7.0 using modified Schaeffer’s Medium in a BSL-3 Enhanced facility within the Galveston National Laboratory, UTMB, purified aseptically by washing in sterile molecular grade water, and then by density gradient centrifugation in 58% MD-76 (Mallinckrodt, Inc., St. Louis, MO). The spores had a germination index of 1.0 and 99.9% optical refractility as determined by phase contrast microscopy. The purified spores were stored in 1% phenol and 0.05% Tween 20 at 4 °C. Mouse macrophage cell line J774A.1 was purchased, maintained and grown as specified (American Type Culture Collection, Manassas, VA).

### Construction of Ty21a-PA-01

A codon optimized gene for PA fused to the Hly (hemolysin) secretion machinery under the lipoprotein gene (*lpp*) promoter from plasmid pLpp-PA_op_-hlyA_s_^34^ was subcloned into pMD-TV,^50^ resulting in a recombinant plasmid L52. L52 was the template DNA for polymerase chain reaction (PCR) amplification; resulting in a product containing the 3′ ~500 bp of *tviD* gene (encoding Vi polysaccharide biosynthesis protein of *S*. Typhi), a flippase-recognization target site-flanked kanamycin resistance cassette (Kan^r^, 1,393 bp), the PA expression cassette (6,130 bp), and ~1000 bp of the *vexA* gene (encoding Vi polysaccharide export protein of *S*. Typhi). The PCR fragment was integrated into the Ty21a chromosome using the λ Red recombination-based recombineering technology.^50, 57^ The Kan^r^ selectable marker was deleted from the chromosomal integrants by transforming cells with plasmid pCP20 and selecting for Kan^s^ transformants as described.^50, 58^ Chromosomal integration and selection marker eviction were confirmed by genomic PCR analysis. We confirmed production of PA by western blot analysis.

### Seed bank generation and characterization

Ty21a-PA-01 was grown in CY medium at 37 °C, as described for Vivotif,^46^ with aeration to late log phase. A seed bank of 200, 1-mL vials was produced and maintained at −80 °C. Two vials were chosen randomly, and characterized by microbiological, immunological, phenotypic analyses, and rDNA sequencing for strain identification (Table 1). The integrity of the 6,130 bp antigenic insert and the entire genome was confirmed by whole genome sequencing. Production of extracellularly secreted PA in culture supernatants and agar-grown colonies was confirmed by western blot analysis using mouse monoclonal anti-PA antibody (Alpha Diagnostic International, San Antonio, TX), as described.^34^

### Manufacturing of foam-dried vaccine

Ty21a-PA-01 was grown in CY medium supplemented with 0.02% galactose to early stationary phase.^46^ The organisms were harvested, resuspended in CY medium, and mixed 1:1 with a 2× FPA cocktail in CY medium to achieve a final concentration of 25% trehalose (w/w), 5% gelatin (w/w), 0.5% methionine (w/w), and 0.34% potassium phosphate, pH 8.0. Five mL aliquots of the Ty21a-FPA formulation were dispensed into 100-mL Schott vials (Allergy Laboratories, Oklahoma City, OK). Foam drying was performed using a Virtis Advantage XL-70 lyophilizer with the following program: Decreasing pressure from 760 Torr to 80 Torr over 15 min at 10 °C, holding at 80 Torr for 45 min, decreasing pressure to 5 Torr over 20 min, and a further decrease to 1 Torr at 22 °C with holding for 2 h and further reduction in pressure to < 100 m Torr and holding at 15 °C for 48 h. Stoppering was performed in the lyophilizer after argon purging. Residual water content was determined using the Karl Fischer method on a Mettler–Toledo Stromboli balance.

### Stability of foam-dried vaccine

Foam-dried vaccine was stored at 4 °C, ambient temperature, and 37 °C, with calibrated incubator temperatures ranging between 2–8, 20–25, and 36–38 °C, respectively. At indicated time points, a sample was removed for assessment that included viable counts and residual water content. Viable counts were performed by reconstituting foam dried material in an equivalent volume of sterile water to the original pre-foam dried state (mL/mg) and quantitated by plating on tryptic soy agar and scoring for colony forming units (CFU)/mg in the foam dried product after overnight incubation at 37 °C.

### Animal immunization

All animal experiments were performed according to guidelines in the *Guide for the Care and Use of Laboratory Animals* (NIH, Bethesda). Sterne strain-susceptible female A/J mice, 6–8 weeks old, and female New Zealand White (NZW) rabbits, ~6 weeks old (1.4–1.8 kg), were purchased from Jackson Laboratory (Bar Harbor, ME), and Harlan Laboratories (Houston, TX), respectively, and maintained at Bioqual (Rockville, MD). They were immunized and assessed according to a protocol approved by the Bioqual Laboratory Animal Care and Use Committee. Twenty mice were immunized by the IP route three times at 2-week intervals with 5 × 10^7^ CFU of Ty21a-PA-01 in 0.5 mL phosphate-buffered saline (PBS). Ten control mice received 5 × 10^7^ CFU of Ty21a IP in the same volume and regimen. Ten rabbits were immunized IP 4 times at 4-week intervals with 1 × 10^9^ CFU of Ty21a-PA-01 or Ty21a in 1.0 mL of PBS. The Ty21a-PA-01 used for animal immunizations had been foam-dried and stored at 4 °C for 3 months prior to the mouse and 15 months prior to the rabbit studies.

### Antibody assessment

Recombinant proteins (rPA and rLF) were obtained from the Biodefense and Emerging Infections Research Resources Repository (BEI, Manassas, VA) or purchased from List Biological Laboratories (Campbell, CA). Antibody titers against PA were determined by enzyme-linked immunosorbent assay (ELISA). Plates were coated with rPA at 2.0 ng/µL in a volume of 50 μL/well and incubated overnight at 4 °C. Plates were then washed three times with 1 × wash solution (KPL, Gaithersburg, MD), and blocked with 1% bovine serum albumin (KPL) containing 1% non-fat dry milk for 1 h at 37 °C. Serially diluted samples were added, and incubated at 37 °C for 1 h and washed as before. Alkaline phosphatase labeled goat anti-mouse IgG or anti-rabbit IgG (KPL) were added at 0.2 ng/µL, plates incubated at 37 °C for 1 h, washed, and developed with Phosphatase substrate (KPL). Data were collected using Softmax 5.0, and fit to a 4-parameter logistic curve. Results were reported as the serum dilution at which the optical density was 1.0 (OD 1.0).

The lethal toxin-neutralizing assay (TNA) titer was determined as described^34^ except J774A.1 murine macrophage cells were used. Briefly, the serially diluted sera were pre-incubated with toxin mixture (PA and LF at 5:4 ratio) at 37 °C for 30 min before adding to J774A.1 cells in 96-well plates. Subsequently, 3-(4,5-dimethylthiazol-2-yl)-2,5-diphenyltetrazolium bromide (MTT, ATCC), 25 µL/well, was added to the cells followed by 1 h of incubation before addition of 100 µL/well of the solubilizing buffer to lyse macrophages. After 10 min, the OD_562_ was read. The toxin neutralizing titer was defined as serum dilution at which 50% of macrophages were killed.

### Sterne spore challenge of A/J mice

Mice were shipped to UTMB after all immunizations. The LD_50_ for Sterne spores was determined in naïve mice of similar age and body weight. All mice were challenged 2 weeks after vaccine dose 3 with an intended target of 5 LD_50_ of *B. anthracis* Sterne spores by nasal instillation. Mice were anesthetized by isoflurane and suspended vertically using monofilament fishing line looped behind the upper incisors and connected to a vertical support platform. An aliquot (20 μL) containing the desired number of spores was placed at the anterior of each naris, and the animals were allowed to inhale the spores for 2–3 min. Then, 10 μL sterile PBS was instilled to wash any non-adherent spores from the nasal cavity into the lungs. Each mouse was calculated to have received 2.8 × 10^5^ spores, corresponding to 4.9 LD_50_. Survival was monitored twice daily for 21 days.

### Ames spore challenge of New Zealand white rabbits

Rabbits were shipped to UTMB after the third immunization at Bioqual. The fourth immunization was administered at UTMB. The LD_50_ for Ames spores was determined in naïve rabbits of similar age and body weight. Two weeks after vaccine dose 4, rabbits were randomly placed into four groups for whole body Ames spore aerosol challenge. The nebulizer for each aerosol run contained a suspension of *B. anthracis* Ames spores (3.5 × 10^9^ CFU/mL), and the mean Sf (spray factor) of the four aerosol runs was 3.9 × 10^−7^ ± 2 × 10^−6^. The presented lung dose/rabbit was based on aerosol sampling during aerosolization and calculated to be 2.0 × 10^7^ CFU of *B. anthracis* Ames spores/animal, corresponding to 200 LD_50_. Survival was monitored twice daily for 21 days.

### Data availability statement

The authors declare that all data supporting the findings of this study, including its supplementary information files, are available from the corresponding author upon reasonable request.
